# Evaluation of the Antitumor Effects of BPR1J-340, a Potent and Selective FLT3 Inhibitor, Alone or in Combination with an HDAC Inhibitor, Vorinostat, in AML Cancer

**DOI:** 10.1371/journal.pone.0083160

**Published:** 2014-01-08

**Authors:** Wen-Hsing Lin, Teng-Kuang Yeh, Weir-Torn Jiaang, Kuei-Jung Yen, Chun-Hwa Chen, Chin-Ting Huang, Shih-Chieh Yen, Shu-Yi Hsieh, Ling-Hui Chou, Ching-Ping Chen, Chun-Hsien Chiu, Li-Chun Kao, Yu-Sheng Chao, Chiung-Tong Chen, John T.-A. Hsu

**Affiliations:** 1 Institute of Biotechnology and Pharmaceutical Research, National Health Research Institutes, Zhunan, Taiwan; 2 Department of Biological Science and Technology, National Chiao Tung University, Hsinchu, Taiwan; Emory University, United States of America

## Abstract

Overexpression or/and activating mutation of FLT3 kinase play a major driving role in the pathogenesis of acute myeloid leukemia (AML). Hence, pharmacologic inhibitors of FLT3 are of therapeutic potential for AML treatment. In this study, BPR1J-340 was identified as a novel potent FLT3 inhibitor by biochemical kinase activity (IC_50_ approximately 25 nM) and cellular proliferation (GC_50_ approximately 5 nM) assays. BPR1J-340 inhibited the phosphorylation of FLT3 and STAT5 and triggered apoptosis in FLT3-ITD^+^ AML cells. The pharmacokinetic parameters of BPR1J-340 in rats were determined. BPR1J-340 also demonstrated pronounced tumor growth inhibition and regression in FLT3-ITD^+^ AML murine xenograft models. The combination treatment of the HDAC inhibitor vorinostat (SAHA) with BPR1J-340 synergistically induced apoptosis via Mcl-1 down-regulation in MOLM-13 AML cells, indicating that the combination of selective FLT3 kinase inhibitors and HDAC inhibitors could exhibit clinical benefit in AML therapy. Our results suggest that BPR1J-340 may be further developed in the preclinical and clinical studies as therapeutics in AML treatments.

## Introduction

Acute myeloid leukemia (AML) is the most common hematologic malignancy in adults with a high incidence rate and low survival probability [1], [2], [3]. AML progresses rapidly due to the rapid growth of abnormal white blood cells that accumulate in the bone marrow and interfere with the production of red blood cells, platelets, and normal white blood cells. If left untreated, AML is usually fatal within weeks or months after diagnosis. FLT3 (FMS-like tyrosine kinase 3), a cell surface receptor belonging to the class III receptor tyrosine kinase family, plays a pivotal role in the differentiation and survival of the hematopoietic stem cells in bone marrow [4], [5]. *FLT3* is one of the most commonly mutated genes in AML [6], [7]. Activating FLT3 mutations, FLT3-ITD (an internal tandem duplication mutation in the juxtamembrane domain) and FLT3-TKD (a missense mutation within the kinase domain), are frequently observed in approximately 30% of adult AML patients [8], [9], [10], [11]. FLT3-activating mutantions critically regulate leukemic transformation by accelerating proliferation and suppressing apoptosis and are significantly associated with poor prognosis [12], [13]. These findings highlight FLT3-ITD and FLT3-TKD as highly attractive therapeutic targets for drug development in human AML.

There are now several classes of small molecule FLT3 inhibitors that have entered clinical trials. However, effective drugs have not yet been identified in clinics [14], [15], [16]. Although these inhibitors have demonstrated promising anti-cancer activity in *in vitro* and *in vivo* preclinical models, clinically positive responses in AML patients receiving single-agent FLT3 inhibitors are limited due to the transient reduction of peripheral blasts but not bone marrow blasts or the occurrence of inhibitor-resistant FLT3 mutations in patients [17], [18], [19], [20]. Therefore, combinatorial strategies of FLT3 inhibitors and other chemotherapeutic agents may be beneficial approaches to improve FLT3 inhibitor therapy and to overcome treatment failures [21], [22]. The FLT3 inhibitor CEP-701 (lestaurtinib) combined with standard AML chemotherapeutic agents has the potential to improve clinical outcomes in AML patients [23]. In addition, histone deacetylase inhibitors (HDACi), a class of compounds that can induce cancer cell growth arrest and cell death by altering the acetylation status of both histone and non-histone proteins, can enhance the activity of FLT3 inhibitors on AML cell apoptosis [24], [25], [26]. The HDACi vorinostat (SAHA) exhibits clinical activity in AML; however, its efficacy as a single agent is only moderate [27], [28]. In this study, we report data characterizing the pharmacological profile of a new FLT3 kinase inhibitor, BPR1J-340, and elucidate the possible molecular mechanism of the strongly synergistic effects in combination with SAHA in FLT3-ITD^+^ cells.

The BPR1J-340 compound exhibits potent FLT3 inhibitory activity, with a 50% inhibitory concentration (IC_50_) of 25±5 nM and growth inhibitory effects on FLT3-ITD^+^ leukemia MOLM-13 and MV4;11 cells with a GC_50_ value of 3.4±1.5 and 2.8±1.2 nM, respectively. The IC_50_ values were approximately 1 nM against FLT3-ITD and 1 nM against STAT5 phosphorylation in MV4;11 cells. In addition, BPR1J-340 exhibits favorable pharmacokinetic properties and significant anti-tumor activity in FLT3-ITD murine xenograft models. The combination of the HDAC inhibitor SAHA with BPR1J-340 exhibits strongly synergistic anti-leukemia effect in FLT3-ITD+ cells. These results highlight the therapeutic potential of BPR1J-340 and SAHA in AML and support its preclinical or clinical development.

## Materials and Methods

### Chemicals and reagents

The FLT3 inhibitors, BPR1J-340 and AC220, were synthesized by our laboratory. The histone deacetylase inhibitor vorinostat (SAHA) was purchased from SelleckBio (Houston, TX, USA). All inhibitors were dissolved in dimethylsulfoxide (DMSO) at a stock concentration of 10 mM. The anti-FLT3 (sc-480, Santa Cruz Biotechnology, Santa Cruz, CA, USA), anti-pFLT3-Tyr591 (#3461, Cell Signaling Technology, Beverly, MA, USA) anti-STAT5 (#9363, Cell Signaling Technology), anti-pSTAT5–Tyr694 (#9351, Cell Signaling Technology), anti-cleaved poly ADP-ribose polymerase (PARP) (#9542, Cell Signaling Technology), anti-Mcl-1 (#4572, Cell Signaling Technology), anti-caspase 3 (#9662, Cell Signaling Technology) and anti-β-actin (Gtx110546, GeneTex, Irvine, CA, USA) antibodies were purchased for Western blotting analysis. The preparation of recombinant proteins, FLT3 (residues Y567-S993), VEGFR1 (residues R781-I1338) and VEGFR2 (residues V789-V1356), for biochemical kinase assay was described previously [29]. The VEGFR3 (residues M800-Y1363) proteins were purchased from Upstate (Billerica, MA, USA).

### Cell culture

RS4;11, MV4;11, U937 and K562 cells were obtained from American Type Culture Collection (ATCC, Manassas, VA, USA). MOLM-13 cells were purchased from the Deutsche Sammlung von Microorganismen und Zellkulturen GmbH (DSMZ, Braunschweig, Germany). All leukemic cell lines were grown in RPMI 1640 (Invitrogen, USA) with 10% fetal bovine serum (FBS) (Fisher Scientific, Pittsburgh, PA, USA). The HEK293T and FLT3-transfected HEK293T cells were cultured in DMEM (Invitrogen, USA) medium with 10% fetal bovine serum (FBS). The other 14 non-leukemic cell lines, HCC827, H1975, H1650, H2228, NCIH460, A431, HCT-116, MKN45, MiaPaCa-2, RT4, MCF-7, Huh7, Hep3B, and Detroit 551, were cultured in medium according to the ATCC recommendations.

### In vitro kinase activity assay

The FLT3 and VEGFR1/2 Kinase-Glo kinase assays were performed as reported by our earlier study [30]. The VEGFR3 activity analysis was conducted using the same protocol described in the VEGFR1/2 assay. The kinase inhibition profiling and inhibitory activity of FLT3-D835Y, CSF1R, and TRKA were determined by Invitrogen SelectScreen® kinase profiling service (Carlsbad, California, USA).

### Cell viability and cell growth assays

Cell viability was assessed with an MTS assay as carried out as the method described previously [31]. Cells were seeded in 96-well plates at a density of 10,000 cells per well for 16 hours and then treated with vehicle or various concentrations of compound in medium. The change in viable cells was quantitated using the MTS method (Promega, Madison, WI, USA) according to the manufacturer's recommended protocol or by cell counting under a microscope. The GC_50_ value was defined as the amount of compound that caused a 50% reduction in cell viability in comparison with DMSO-treated (vehicle) control and was calculated using Prism version 4 software (Graph-Pad Software, Inc., San Diego, CA, USA). The data are presented as the mean ± S.D. from three independent experiments. Cell growth were measured by trypan blue dye exclusion method. Cells (1×10^4^ mL^−1^) were cultured in 12-well plates for 24 hours and then treated with different amounts of test compound and the same volumes of vehicle for 72 hours. The number of viable cells was counted by trypan blue dye staining using a hemocytometer at the indicated time point after drug treatment. The result was expressed as the mean ± S.D. from triplicate determinations.

### Western blotting

Cells were lysed in lysis buffer (50 mM Tris, pH 8.0, 150 mM NaCl, 1% Triton X-100, 0.5% sodium deoxycholate, 0.1% SDS, 1 mM sodium orthovanadate, 1 mM PMSF, and 1 mM DTT). Protein lysates were resolved by SDS-PAGE and transferred onto a polyvinylidene difluoride (PVDF) membrane (Millipore, Bedford, MA, USA). The membranes were immunoblotted with appropriate antibodies and detected using the SuperSignal reagent (Pierce, Rockford, IL, USA) followed by exposure to X-ray film.

### Apoptosis assay

The number of apoptotic cells was determined by fluorescence-activated cell sorting (FACS) analyses using Annexin V-FITC staining. In brief, cells were centrifuged after drug treatment and resuspended in 1× binding buffer containing 2.5 mM of calcium (Ca2+). Cells were incubated with Annexin V-FITC (Biolegend, San Diego, CA) and propidium iodide (PI) (Sigma–Aldrich, St. Louis, MO) in the dark at room temperature for 20 minutes. Next, samples were reconstituted with 1× binding buffer and subjected to flow cytometry FACS Calibus (BD Bioscience, Franklin Lakes, NJ) to analyze the Annexin V-positive population using CellQuest Pro (BD Bioscience) and FlowJo (Treestar, Inc., San Carlos, CA).

### Pharmacokinetic analysis of BPR1J-340

The uses of animals were approved by The Institutional Care and Use Committee of the National Health Research Institutes. Briefly, male Sprague-Dawley rats weighing 300–400 g each were obtained from BioLASCO, Taiwan Co., Ltd., Ilan, Taiwan. One day prior dosing, rats were surgically prepared with a jugular-vein cannula and fasted overnight (∼18–20 hr). Water was available *ad libitum*. Food was provided at 4 hours after dosing. Single 1.5 mg/kg intravenous dose of BPR1J-340, as a PEG400/water (80/20, v/v) solution, was separately administered to groups of 3 rats each via the jugular-vein cannula. At 0 (prior to dosing), 2, 5, 15 and 30 min. and at 1, 2, 4, 6, 8 and 24 hr after dosing, a blood sample was collected. Plasma was separated from the blood by centrifugation (14,000 g for 15 min at 4°C in a Beckman Model AllegraTM 6R centrifuge) and stored in a freezer (−20°C) until analysis. All plasma samples were analyzed by LC-MS/MS. Plasma concentration data were analyzed with non-compartmental method for pharmacokinetic determination.

### Subcutaneously tumor xenograft experiments

Male nude mice (Nu-Fox1nu) of eight weeks old were purchased from BioLasco (Ilan, Taiwan). The nude mice (n = 5∼7 per group) were inoculated subcutaneously with MOLM-13 (1×10^6^ cells/flank). All human cancer cells were determined to be free of Mycoplasma spp before the inoculation. When tumors size reached 100∼200 mm^3^ and a large size of >500 mm^3^, the animals were grouped and treated with the BPR1J-340 (5 and 20 mg/kg, i.v.) or vehicle as control once daily for 5 days per week for two or three weeks. Tumor sizes were measured and calculated with the formula of length×width^2^/2 after the initiation of the treatments. Tumor size and animal body weight were measured twice a week after the tumor cell inoculation. At the end of the study, animals were euthanized by carbon dioxide inhalation followed by cervical dislocation. The significant difference between the treated and vehicle control were analyzed by using one-way *ANOVA* and *Student-Newman-Keuls* test. The level of a statistical significance was set at *p*<0.05.

## Results

### In vitro kinase profiling of BPR1J-340

**Figure 1:**
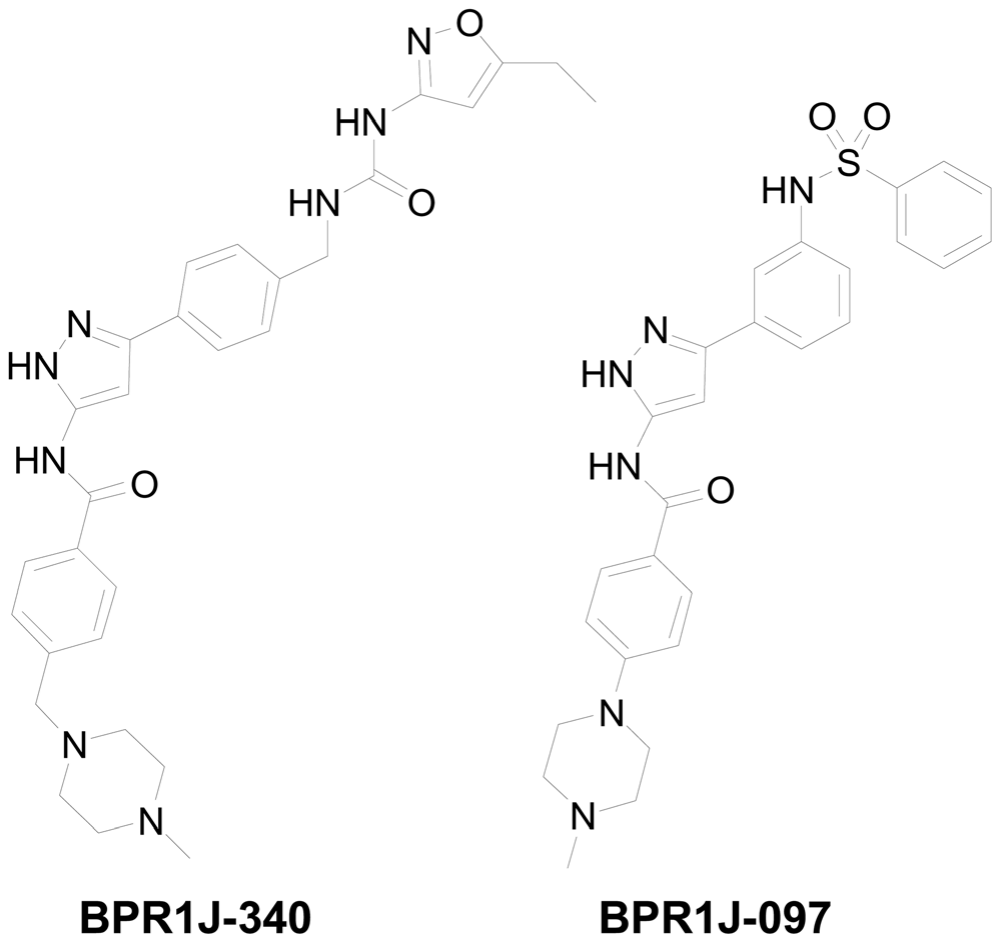
Chemical structure of BPR1J-340 and BPR1J-097. BPR1J-340: *N*1-(3-4-[([(5-ethyl-3-isoxazolyl)amino]carbonylamino)methyl]phenyl-1*H*-5-pyrazolyl)-4-[(4-methylpiperazino)methyl]benzamide. BPR1J-097: *N*1-(3-3-[(phenylsulfonyl)amino]phenyl-1*H*-5-pyrazolyl)-4-(4-methylpiperazino)benzamide.

BPR1J-340, a urea-substituted 3-phenyl-1*H*-5-pyrazolylamine-based compound, was designed through chemical modification of sulfonamide series of derivatives (BPR1J-097 series) by a structure-activity relationship (SAR) study (Fig. 1) [31], [32]. For kinase inhibition specificity screening, BPR1J-340 was tested against 59 protein kinases covering the major oncogenic kinases of human protein kinome. This kinase inhibition profile revealed that BPR1J-340 is a highly selective kinase inhibitor and most of tested kinases were not significantly inhibited at the concentration of 100 nM (Table S1). Subsequently, the most potent kinases from the 59 kinases screening were further evaluated. As shown in Table 1, BPR1J-340 potently inhibited wild-type FLT3 (IC_50_ = 29±5 nM, as compared to ABT869 with an IC_50_ of 38±3 nM,), mutant FLT3-D835Y (IC_50_ = 19 nM), CSF1R (FMS) (IC_50_ = 78 nM), KDR (VEGFR2) (IC_50_ = 28±9 nM), and FLT4 (VEGFR3) (IC_50_ = 29±7 nM). In addition, BPR1J-340 inhibited TRKA, which is associated with the growth and metastasis of breast cancers, with an IC_50_ value of 8 nM. Given the high similarity of the ATP binding pockets of FLT3 and the Aurora kinases, the inhibitory activity toward Aurora A and Aurora B were measured. The IC_50_s of BPRJ-340 were determined to be 1,040 nM and 1,344 nM for Aurora A kinase and Aurora B kinase, respectively. Taken together, BPRJ-340 is a selective kinase inhibitor with *in vitro* activity against FLT3, VEGFR2, VEGFR3 and TRKA receptor tyrosine kinases.

**Table 1:** Specificity of kinase inhibition of BPR1J-340.

Kinase	Percentage of inhibition^a^	IC50, nM
FLT3	97	29±5^b^
TRKA	89	8*
FLT3-D835Y	78	19*
CSF1R (FMS)	73	78*
KDR (VEGFR2)	69	28±9^b^
FLT4 (VEGFR3)	45	29±7^b^
FLT1 (VEGFR1)	15	164±24^b^

^a^ Inhibition of kinase at 100 nM, carried out by Invitrogen SelectScreen® kinase profiling service.

^b^ IC50 determination was performed by kinase-Glo assay.

IC_50_ determination was performed by Invitrogen SelectScreen® kinase assay.

Note: IC_50_ of DBPR1J-340 against multiple oncogenic kinases; the 7 most potent kinases from the 59 kinases screened are shown.

### BPR1J-340 inhibits the proliferation of FLT3-ITD^+^ cells

After the selective and potent growth inhibitory effects of BPR1J-340 were demonstrated in FLT3-ITD-bearing leukemic cells, the cell proliferation inhibitory activity was tested in a panel of leukemic and non-leukemic cells. BPR1J-340 inhibited the cellular proliferation of FLT3-ITD^+^ cells (MOLM-13 and MV4;11 FLT3-dependent cell lines) with an GC_50_ of approximately 5 nM. Cells that were not dependent on FLT3 signaling for growth, including RS4;11, U937, and K562 leukemic cells [33], [34], [35], and the non-leukemic cell lines tested were either weakly inhibited or were not inhibited by BPR1J-340 (Table 2). The growth of the FLT3-independent RS4;11 cell line which contains wild-type FLT3 was only weakly inhibited by BPR1J-340 with an GC_50_ value of 770±360 nM. The sensitivity toward BPR1J-340 varies between the FLT3-dependent cells MOLM-13/MV4;11 and FLT3-independent cells RS4;11, suggesting that the FLT3 signaling pathway in FLT3-ITD+ cells is almost completely disturbed by BPR1J-340. Furthermore, our results suggest that BPR1J-340 could inhibit FLT-ITD activity by *in vitro* biochemical and cellular assays. Overall, BPR1J-340 is a potent and highly selective inhibitor for the proliferation for FLT3-driven cells.

**Table 2:** Anti-proliferative activity of BPR1J-340 against a panel of tumor cell lines.

Cell lines	Proliferation GI50, nM
**Leukemias**	
MOLM-13	3.4±1.5
MV4;11	2.8±1.2
RS4;11	770±360
U937	2,400±780
K562	6,370±2,070
**Non-Leukemias**	
HCC827	2,470±380
H1975	2,610±270
H1650	3,725±977
H2228	2,520±420
NCIH460	13,160±2,840
A431	6,570±680
HCT-116	9,330±2,320
MKN45	3,580±1,210
MiaPaCa-2	9,960±570
RT4	7,030±1,510
MCF-7	11,300±1,110
Huh7	9,740±1,680
Hep3B	8,310±490
Detroit551	8,340±680

### BPR1J-340 inhibits FLT3-STAT5 signaling

**Figure 2:**
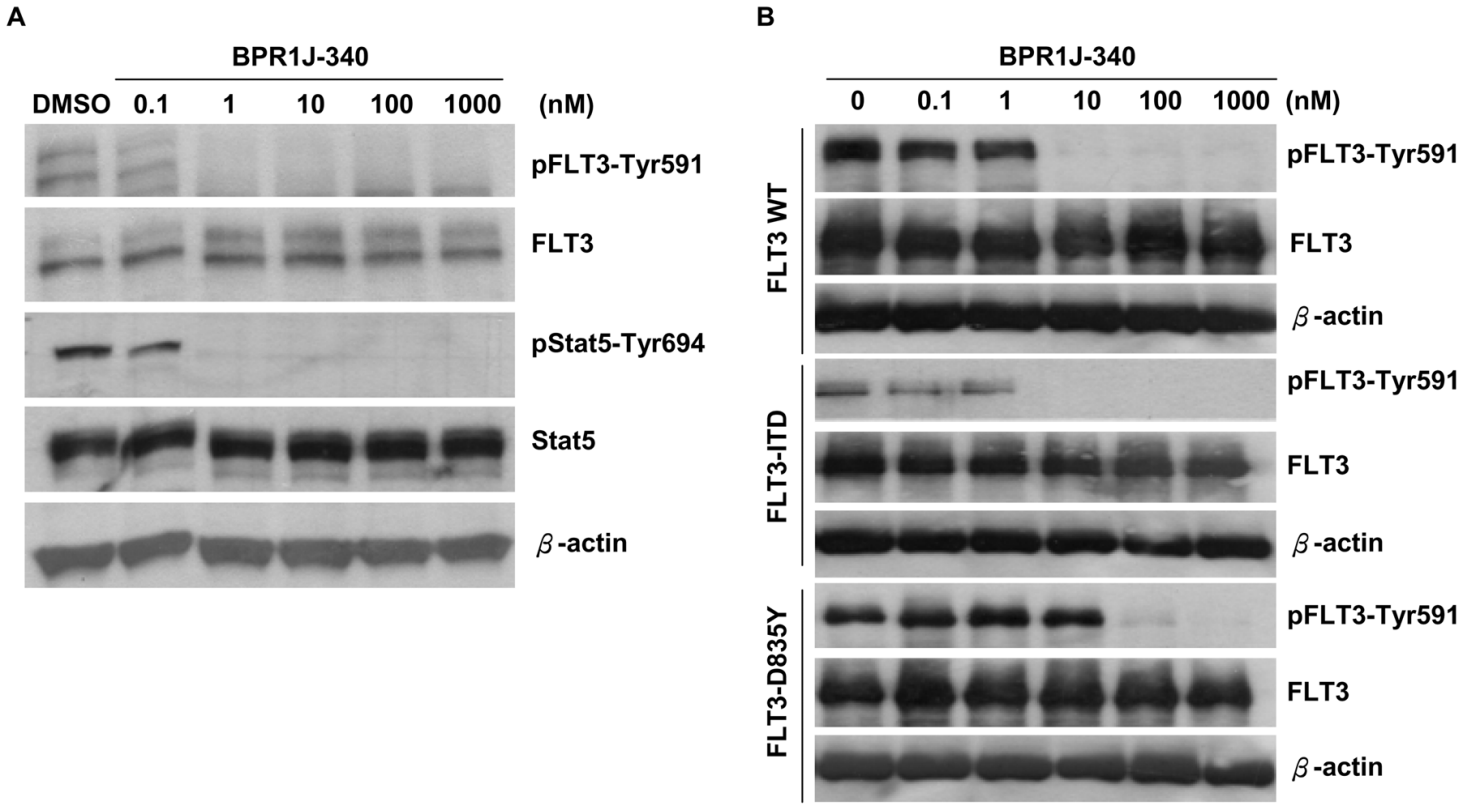
BPR1J-340 inhibits FLT3-dependent signaling. (**A**) MV4;11 cells were treated with BPR1J-340 at the indicated concentrations for 1 hour. The phosphorylation status of FLT3 and STAT5 were evaluated by Western blot analysis. (**B**) HEK 293T cells were transfected with FLT3-WT-, FLT3-ITD-, or FLT3-D835Y-expressing plasmids for 24 hours and then incubated with various concentrations of BPR1J-340 for 1 hour. The FLT3 phosphorylation status in the transfected cells was evaluated by Western blot analysis.

To address whether BPR1J-340 was able to inhibit the FLT3 signaling pathway in FLT3-driven cells, MV4;11 cells were treated with different concentrations of BPR1J-340 for 1 hour, and the phosphorylation status of FLT3 and STAT5 (Signal Transducer and Activator of Transcription 5), a critical down-stream modulator that plays a major role in FLT3-ITD signal transduction for cell expansion and survival, was examined by Western blot analysis. As shown in Figure 2A, BPR1J-340 suppressed the phosphorylation of FLT3 and STAT5 in a dose-dependent manner with an IC_50_ value of approximately 1 nM. To investigate the difference in sensitivity between FLT3-WT and the activating mutants FLT3-ITD and FLT3-D835Y to BPR1J-340, HEK293T cells engineered to express FLT3-WT or mutants FLT3 (FLT3-ITD, FLT3-D835Y) were analyzed [31]. The HEK293T-FLT3 cells were treated with BPR1J-340 at various concentrations for 1 hr, and FLT3 ligand (50 ng/mL) was added for 5 min to prepare the cell lysate for detection of changes in FLT3 phosphorylation by Western analysis. As shown in Figure 2B, the phosphorylation of all the FLT3-WT, FLT3-ITD and FLT3-D835Y was inhibited by BPR-1J340, with IC_50_ values of 10 to 100 nM. Taken together these data demonstrate that BPR-1J340 inhibits cellular FLT3 phosphorylation and modulates the FLT3 signaling pathway, particularly the FLT3-ITD pathway.

### BPR1J-340 induces apoptosis in FLT3-ITD expressing cells

**Figure 3:**
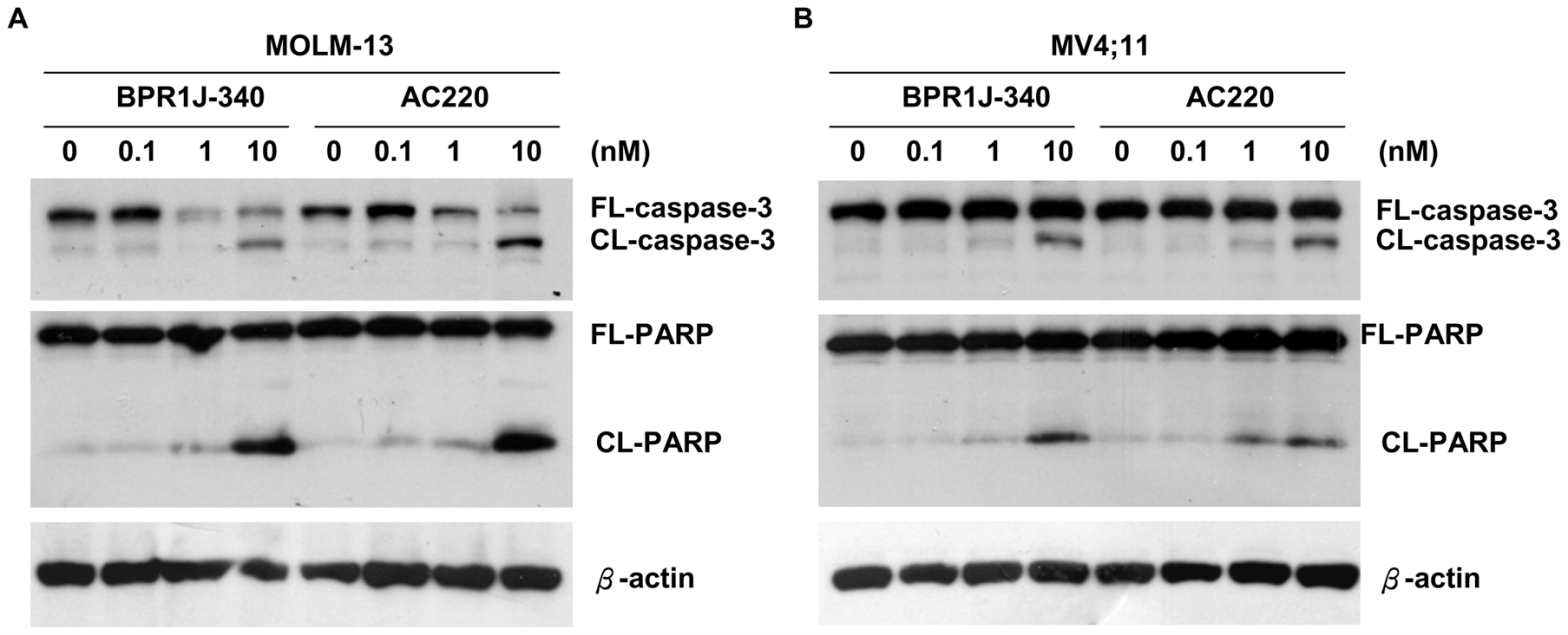
BPR1J-340 induces apoptosis in MOLM-13 and MV4;11 cells. Western blotting revealed that BPR1J-340 was able to induce apoptosis in FLT3-ITD-driven FLT3-ITD^+^ AML cells. MOLM-13 (**A**) and MV4;11 (**B**) cells were treated with BPR1J-340 at the indicated concentrations for 24 hours, and the cell lysates were then subjected to Western blot analysis using antibodies against caspase-3 and PARP (poly-ADP-ribose polymerase). (full length caspase-3 (FL-caspase-3), cleavage of caspase-3 (CL-caspase-3), full length poly(ADP-ribose) polymerase (FL-PARP), cleavage of PARP (CL-PARP)).

As the inhibition of FLT3 signaling results in a loss of growth potential and an induction of apoptosis in FLT3-ITD expressing cells, the effects of BPR1J-340 on cell apoptotic response in FLT3-ITD^+^ cells was investigated. The MOLM-13 and MV4;11 cells were treated with BPR1J-340 at different concentrations for 24 hours and analyzed for the induction of apoptosis using active caspase-3 and cleaved PARP (poly-ADP-ribose polymerase) analysis. The ability of BPR1J-340 to induce apoptosis is evident as shown in Figure 3 where significant cleavage of caspase-3 (active caspase-3) and PARP (active PARP) was observed in MOLM-13 and MV4;11 cells treated with BPR1J-340 at 10 nM.

### SAHA in combination with BPR1J-340 enhances cytotoxicity against FLT3-ITD^+^ expressing cells

**Figure 4:**
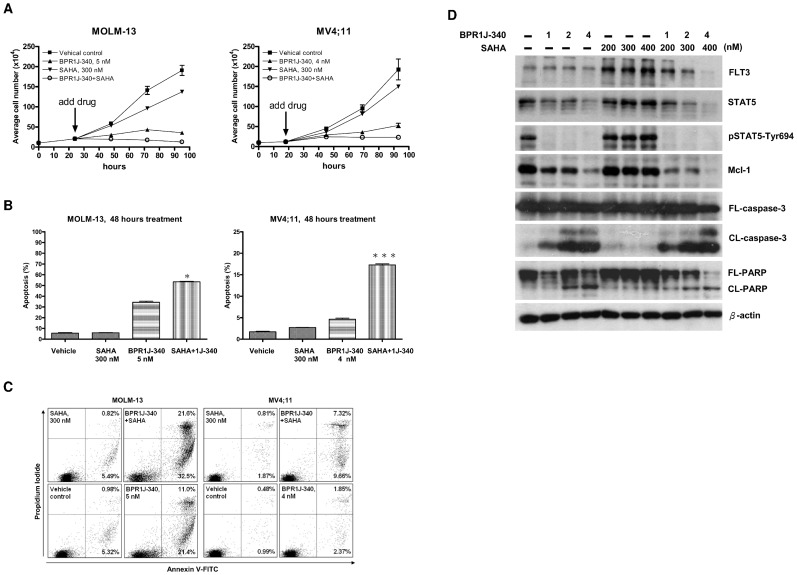
The effect of BPR1J-340 and SAHA versus drug combination on AML FLT3-ITD+ cells. MOLM-13 or MV4;11 cells were seeded at an initial concentration of 1×10^5^ cell mL^−1^ for 24 hours and then treated with BPR-1J340 either alone or in combination with SAHA in the indicated concentrations. (**A**) Viable cells were counted after staining with trypan blue dye at indicated time point (**B**) At 48-hours after treatment, cells were stained with FITC-labeled annexin V and propidium iodide and percentages of apoptotic cells were analyzed by flow cytometry. (**C**) The representative flow cytometry histograms of assays of annexin positive apoptotic cells at 48 hours drug treatment (**D**) MOLM-13 cells were treated with drugs for 20 hours, and then followed by Western blot analysis to assess changes in protein levels with the indicated antibodies. Statistical analysis was performed using Student's *t*-test. *Indicates significant difference compared to vehicle control (* p<0.05, * * * p<0.01). Data shown are representative of multiple independent experiments.

Some histone deacetylase inhibitors (HDACi) will enhance the cytotoxic activity of FLT3 inhibitor on FLT3-ITD^+^ cell via degradation of FLT3-ITD and STAT5 [24], [26], [36], [37]. To determine whether SAHA, an HDACi, can synergize with BPR1J-340 to enhance cytotoxicity in FLT3-ITD expressing cells by interfering with the FLT3-ITD and STAT5 axis, the combined effects of SAHA and BPR1J-340 on cytotoxicity were examined. The cell growth rate of MOLM-13 and MV4;11 with SAHA and BPR1J-340 combinational treatment was decreased compared with the single-drug treatment (Fig. 4A). Next, we examined the SAHA treatment effect on BPR1J-340-induced apoptosis in FLT3-ITD-expressing cells. Sole treatment with SAHA (at 300 nM) for 48 hours did not increase the rate of apoptotic cells in control population 10%, whereas SAHA/BPR1J-340 co-treatment resulted in a greater induction of apoptosis (increased 20% in MOLM-13 and 12% in MV4;11 cells, respectively) as compared with BPR1J-340 drug treatment alone (Fig. 4B and Fig. 4C). These data suggested that SAHA enhance BPR1J-340-induced apoptosis in FLT3-ITD^+^ cells. We further elucidated the protein levels of FLT3-ITD and STAT5 in this SAHA/BPR1J-340 co-treatment enhanced cytotoxicity. We treated MOLM-13 cells with SAHA, BPR1J-340, or their combination for 20 hours. Compared with single-drug treatment, the combination of SAHA and BPR1J-340 remarkedly decreased the protein levels of FLT3-ITD and STAT5 (Fig. 4D). Furthermore, BPR1J-340/SAHA treatment profoundly reduced Mcl-1 (myeloid cell leukemia-1, an anti-apoptotic member of the BCL-2 family) protein levels in FLT3-ITD^+^ cells (Fig. 4D). This results of Mcl-1 reduction may explain the enhanced cytotoxicity even the STAT5 phosphorylation at Tyr694 was completely block by BPR1J-340 (Fig. 4D) Mcl-1 plays an essential role in resistance to chemotherapy in AML cells [38]. Thus, targeting MCL-1 using SAHA/BPR1J-340 may be a promising strategy to overcome drug resistance in FLT3-ITD-positive AML. Our results suggest that the reduction in the total protein levels of FLT3-ITD, STAT5 and Mcl-1 by SAHA addition provides a possible mechanism to explain the enhanced cytotoxicity with SAHA/BPR1J-340 coadministration.

### Pharmacokinetic parameters of BPR1J-340

**Figure 5:**
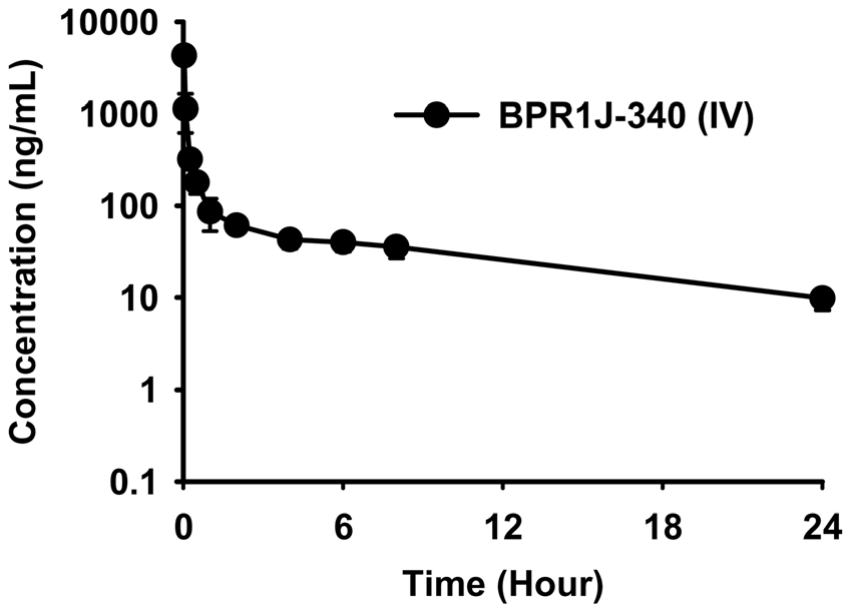
Pharmacokinetic profile of BPR1J-340 in rats. A single intravenous bolus dose (1.5 mg/kg) of BPR1J-340 was administered to adult male Sprague-Dawley rats (n = 3). Data illustrate mean values (n = 3) ± S.D. of plasma concentrations of BPR1J-340 at each timepoint.

To evaluate the pharmacokinetic properties of BPR1J-340, we measured the plasma concentration of BPR1J-340 over a 24-hr period after a single intravenous administration (Fig. 5 and Table 3). After a 1.5 mg/kg intravenous dose administered to Sprague-Dawley rats, BPR1J-340 achieved a maximum plasma concentration of 7.9 µM (4,296 ng/mL) at 2 min after dosing. The plasma concentration exceeded 80 ng/mL for 6 hours and remained steady at 9.9 ng/mL (C_24 hour,_ 18 nM) after 24 hour dosing. Therefore, the plasma concentration of BPR1J-340 reaches sufficient levels to inhibit proliferation and induce apoptosis of FLT3-ITD^+^ cells based on cellular experiments. The total body clearance was 20.4±5.2 mL/min/kg and the volume of distribution at the steady state (Vss) was 10.3±2.5 L/kg for BPR1J-340 in rats. The apparent plasma half-life was approximately 8.8 hr.

**Table 3:** Pharmacokinetic parameters of BPR1J-340 in rats.

	Rat IV (dose: 1.5 mg/kg)			
Compound	T1/2	CL	Vss	AUC_(0–24)_
	(hr)	(mL/min/kg)	(L/kg)	(ng/mL×hr)
**BRP1J-340**	8.8±0.6	20.4±5.2	10.3±2.5	1130±260

### Tumor growth suppressive activity of BPR1J-340

**Figure 6:**
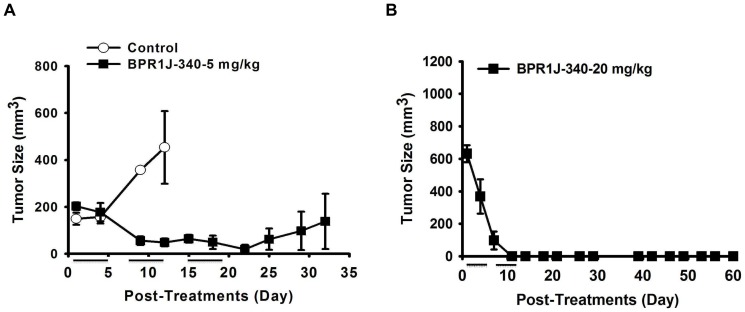
Antitumor activity of BPR1J-340 against FLT3-ITD-driven leukemia tumor growth in nude mice. BPR1J-340 (i.v.) is active against human acute leukemia MOLM-13 tumors growing subcutaneously and can reduce the size of tumors even after the tumors were allowed to grow to a size of >630 mm^3^. (**A**) The in vivo antitumor effect of BPR1J-340 in the MOLM-13 xenograft nude mice model. The growth of the tumor xenograft was inhibited by BPR1J-340 [5 mg/kg, i.v.]; p<0.05 compared with vehicle treatment. (**B**) The subcutaneously growth of a MOLM-13 tumor of large size (>630 mm^3^) in nude mice could be significantly reversed by BPR1J-340 [20 mg/kg, i.v.].

FLT3-ITD^+^ MV4;11 and MOLM-13 xenografts have been widely used to evaluate the *in vivo* efficacy of FLT3 inhibitors against AML [39], [40]. We and other groups have found the MV4;11 xenograft model is noticeably more sensitive to FLT3 inhibitors [30], [31], [39], [41]. To assess the ability of BPR1J-340 to inhibit tumor growth in an AML xenograft model, we therefore selected the MOLM-13 tumor xenograft model. BPR1J-340 was administered intravenously (5 mg/kg qd) on days 1–5 of each week for 3 weeks; by the end of this period the MOLM-13 tumors had reached an average volume of 200 mm^3^. Treatment via this regimen resulted in complete regression of the tumor in all animals by day 22; complete regression (CR) occurred in 4 of 6 treated animals within 31 days of post-treatment observation (Fig. 6A). In this efficacy study, mice with larger tumors (average tumor volume 630 mm^3^, n = 3) were given a single intravenous dose of 20 mg/kg BPR1J-340 on days 1–5 of each week for 2 weeks, which resulted in complete and durable tumor regression with no palpable tumors detected during the 48-day follow up (Fig. 6B). No loss of body weight or mortality was observed in animals treated with any dose of BPR1J-340 (data not shown). These results demonstrate that BPR1J-340 effectively reduces tumor size at intravenous doses of 5 and 20 mg/kg/day; BPR1J-340 is well-tolerated in mice at these effective doses.

## Discussion

Given that the abnormal expression of FLT3 kinase, including amplified or aberrantly activated FLT3, is frequently observed in the blast cells of AML patients, FLT3 represents an attractive therapeutic target of choice for drugs development in AML. To date, several potential FLT3 inhibitors have been developed and examined in AML patients, including lestaurtinib (CEP701) and midostaurin (PKC412) in phase III clinical trials and sunitinib malate (Sutent), sorafenib (Nexavar), quizartinib (AC220), and crenolanib (CP-868596) in phase II trials. However, FLT3 kinase targeting by mono-therapy with current experimental agents does not yield therapeutic benefits in AML patients. It indicated that the aberrant activation of FLT3 and/or drug-resistant FLT3, including pre-existing and acquired drug-resistant mutants, could rarely be fully inhibited by single-agent treatment. Thus, there is a need for the identification of more effective inhibitors of FLT3 and the development of novel therapeutic approaches, including drug combination strategies that target not only FLT3 but also molecules relevant to the FLT3 survival pathway to override current drug resistance. In this study, we demonstrated the efficacy of the novel FLT3 inhibitor BPR1J-340 in various *in vitro* and *in vivo* models of AML and identify synergistic effects with HDACi SAHA on the cytotoxicity of FL3-ITD-expressing cells in *in vitro* analyses.

Previously, we identified a sulfonamide series of 3-phenyl-1*H*-5-pyrazolylamine-based compounds as potent inhibitors of FLT3 such as BPR1J-097 [31]. In continuing to our efforts to develop potent FLT3 inhibitors, we intended to search other series of inhibitors that not only increased the *in vitro* growth-inhibitory effect on AML cells but also prolonged the duration of action *in vivo*. Through rational design, we discovered BPR1J-340, which is a urea series of 3-phenyl-1*H*-5-pyrazolylamine-based FLT3 inhibitor, with effectively inhibits FLT3-WT or FLT3-ITD activity *in vitro* and *in vivo*. Because multiple signaling pathways influence the growth and metastatic potential of tumor cells, many of the inhibitors in clinical development are designed as multi-targeted inhibitors that block a limited number of oncogenic kinases. Thus, the kinase selectivity profiling of BPR1J-340 was performed to identify additional targets in a panel of 59 tested oncogenic kinases. In further biochemical assay, BPR1J-340 demonstrated potent inhibition (20–190 nM) against the angiogenic kinases VEGFR1, VEGFR2, and VEGFR3, which all play an important role in the tumor microenvironment (Table 1). In addition, BPR1J-340 potently inhibited TRKA activity with an IC_50_ value of 8 nM. Taken together, BPRJ-340 is characterized as a selective multi-targeted inhibitor with potent inhibition activity against FLT3-WT, FLT3-D835Y, VEGFR2, VEGFR3, and TRKA. This inhibition profile may allow BPRJ-340 to inhibit tumor growth directly by blocking the aberrant FLT3 signaling pathway and indirectly by targeting tumor angiogenesis. BPR1J-340 may also have clinical potential in tumor driven by abnormally expressed TRKA receptors, which can occur in brain, prostate, pancreatic, and breast cancer [42], [43], [44], [45].

BPR-1J340 inhibited cellular FLT3 phosphorylation and modulated the FLT3 signaling pathway, which resulted in inhibition of proliferation and induction of apoptosis. BPR1J-340 demonstrated potent growth inhibition, predominantly in FLT3-dependent cells (MOLM-13 and MV4;11) but not in FLT3-independent cells. BPR1J-340 inhibited the proliferation of mutant FLT3-ITD^+^ cells with an GC_50_ of 2–6 nM and inhibited FLT3-ITD phosphorylation with an IC_50_ of 10 nM; even the cells transfected with the FLT3-D835Y mutant were also inhibited by BPR1J-340 with an IC_50_ of approximately 100 nM. Consistent with these results, BPR1J-340 effectively induced apoptosis in FLT3-ITD^+^ cells.

HDAC inhibitors may exhibit growth inhibition activity against AML cells and significantly improve the therapeutic efficacy of FLT3 inhibitors [24], [46]. A recent study reported that HDACi LBH589 plus an FLT3 inhibitor (PPKC412, AC220) combination treatment could synergistically induce apoptosis through FLT3-ITD and STAT5 degradation [26]. It also demonstrated that activated caspase-3 (CL-caspase 3) contributes to the degrdation of FLT3-ITD and STAT5 [26]. FLT3-ITD degradation was also reported secondary to HDACi-induced up-regulation of UBCH8 and down-regulation of HSP90 [36], [47]. Mcl-1, an anti-apoptotic protein that promotes survival of FLT3-ITD^+^ cells via STAT5 activation, is down-regulated by FLT3 inhibition [25]. The HDACi SAHA also induced down-regulation of Mcl-1 [48]. Furthermore, Mcl-1 protein is a direct cleavage substrate of activated caspase-3 [49]. We noted that the amount of Mcl-1 correlated with iuduction of activated caspase-3 (Figure 4D). Our results demonstrate that SAHA enhances BPR1J-340 inhibition activity in FLT3-ITD^+^ cells and suggests that the enhancement is due to HDACi-induced reduction of FLT3-ITD, STAT5, and Mcl-1. However, the underlying mechanism of enhanced action by combination treatment remains to be further elucidated.

The maximum achievable plasma concentration of BPR1J-340 after a single 1.5 mg/kg in rat is more than 272-fold above the IC_50_ for FLT3-ITD inhibition in biochemical and cellular assays. Even at 24 hour after the single dosing, the plasma levels of BPR1J-340 were close to the IC_50_ value for inhibition of FLT3-ITD. In addition, the high Vss indicated that the distribution of BPR1J-340 into deep tissue compartments, including tumor tissue, is expected. These pharmacokinetic properties suggest that BPR1J-340 dosing once a day is sufficient for continuous inhibition of FLT3 activity in rats or mice.

To examine whether BPR1J-340 exhibits antitumor activity in vivo, MOLM-13 cells were subcutaneously implanted into nude mice. Our results demonstrated that BPR1J-340 administration resulted in significant tumor regression and tumor shrinkage in this MOLM-13 tumor model. In comparison with sulfonamide BPR1J-97 (1/8 CR, 25 mg/kg) in the same model [31], BPR1J-340 results in a higher CR ratio (67%) at a lower dose (5 mg/kg). These data demonstrated that BPR1J-340 is superior to the sulfonamide compound BPR1J-097 in an *in vivo* efficacy study.

In conclusion, results from this study demonstrate that BPR1J-340 exhibits high potency and excellent selectivity against FLT3 kinase, strong suppression of the FLT3-ITD survival signaling pathway, favorable pharmacokinetic properties, and complete tumor regression in a FLT3-ITD^+^ xenograft model. These data together support further clinical investigation of PR1J-340 in patients with AML. In addition, the BPR1J-340 potentiated the anti-proliferative activity of the HDAC inhibitor SAHA against human leukemia cells. The combination of SAHA and BPR1J-340 should be a good candidate therapy to develop as a treatment in AML and further investigation in clinical study is warranted.

## Supporting Information

Table S1 Kinase inhibition profiles of BPR1J-340. Percentage inhibition was determined by Invitrogen SelectScreen kinase profiling service.
(XLS)
